# The Structure of the T190M Mutant of Murine α-Dystroglycan at High Resolution: Insight into the Molecular Basis of a Primary Dystroglycanopathy

**DOI:** 10.1371/journal.pone.0124277

**Published:** 2015-05-01

**Authors:** Manuela Bozzi, Alberto Cassetta, Sonia Covaceuszach, Maria Giulia Bigotti, Saskia Bannister, Wolfgang Hübner, Francesca Sciandra, Doriano Lamba, Andrea Brancaccio

**Affiliations:** 1 Istituto di Biochimica e Biochimica Clinica, Università Cattolica del Sacro Cuore, Roma 00168, Italy; 2 Istituto di Cristallografia—CNR, Trieste Outstation, Trieste, 34149, Italy; 3 School of Biochemistry, University of Bristol, Bristol, BS8 1TD, United Kingdom; 4 Department of Physics, University of Bielefeld, 33615, Bielefeld, Germany; 5 Istituto di Chimica del Riconoscimento Molecolare—CNR, c/o Università Cattolica del Sacro Cuore, Roma, 00168, Italy; National Institute for Medical Research, Medical Research Council, London, UNITED KINGDOM

## Abstract

The severe dystroglycanopathy known as a form of limb-girdle muscular dystrophy (LGMD2P) is an autosomal recessive disease caused by the point mutation T192M in α-dystroglycan. Functional expression analysis *in vitro* and *in vivo* indicated that the mutation was responsible for a decrease in posttranslational glycosylation of dystroglycan, eventually interfering with its extracellular-matrix receptor function and laminin binding in skeletal muscle and brain. The X-ray crystal structure of the missense variant T190M of the murine N-terminal domain of α-dystroglycan (50-313) has been determined, and showed an overall topology (Ig-like domain followed by a basket-shaped domain reminiscent of the small subunit ribosomal protein S6) very similar to that of the wild-type structure. The crystallographic analysis revealed a change of the conformation assumed by the highly flexible loop encompassing residues 159–180. Moreover, a solvent shell reorganization around Met190 affects the interaction between the B1–B5 anti-parallel strands forming part of the floor of the basket-shaped domain, with likely repercussions on the folding stability of the protein domain(s) and on the overall molecular flexibility. Chemical denaturation and limited proteolysis experiments point to a decreased stability of the T190M variant with respect to its wild-type counterpart. This mutation may render the entire L-shaped protein architecture less flexible. The overall reduced flexibility and stability may affect the functional properties of α-dystroglycan *via* negatively influencing its binding behavior to factors needed for dystroglycan maturation, and may lay the molecular basis of the T190M-driven primary dystroglycanopathy.

## Introduction

Dystroglycan (DG) is composed of two subunits, α and β, that are formed from a unique precursor which undergoes a proteolytic event within the endoplasmic reticulum during the first steps of its maturation pathway [1, 2]. DG represents a widely expressed adhesion complex that plays a crucial role in offering stability to tissues, being at the crossroad between cytoskeleton, plasma membrane and the surrounding extracellular matrix [3, 4]. The DG main role is to bind with high-affinity laminins that are central organizers of the molecular network behind specialized basement membranes surrounding skeletal muscle. Besides, DG may form a plethora of additional interactions with other binding partners sharing laminin globular (LG) domains within epithelia, endothelia and Schwann cells. Indeed, its binding affinity may largely vary based on the degree of glycosylation of its α subunit [5].

DG has been particularly studied within skeletal muscle, a tissue where its α-subunit is found to be highly glycosylated, since a number of neuromuscular diseases involving glycosyltransferases active in muscle have been identified during the last years [6]. The commonly accepted molecular scenario implies that hypoglycosylated DG cannot bind the skeletal muscle laminin-2 [7] with the usual high affinity, which would lead to the reduction of sarcolemma stability at the basis of a wide number of severe, or later onset, muscular dystrophies, defined as secondary dystroglycanopathies [6].

The α-subunit plays multiple important roles in DG, in that i) it is crucially involved in the maturation and posttranslational modification of the precursor; ii) it is highly modified with sugar chains especially in its central region (i.e. the mucin-like portion) from where some essential sugar moieties protrude to bind laminin. The first is based on the noncovalent binding activity of α-DG toward β -DG [8] while for the latter, it seems that the N-terminal domain may be central in recognizing and directing the activity of important modifying enzymes, such as the like-acetylglucosaminyltransferase LARGE, that adds the repeating disaccharide block [-α3-glucuronic acid (GlcA)-β3-xylose (Xyl)-) required for laminin-binding [9] to a phosphorylated mannose of α-DG [10].

We have pioneered studies on α-DG at the molecular level [11], having extensively characterized the structure of its N-terminal domain, which is shaped in two subdomains [12]. The first is a typical Ig-like domain, while the second one is similar to the S6 protein present in the small ribosomal subunit of *T*. *termophilus* [13].

Although a plethora of interesting studies are currently unraveling the structure of the sugar blocks modifying the mucin-like domain of α-DG as well as the exact residues of α-DG involved in these modifications [14–17], no ultimate structural information is currently available on the dystroglycan/laminin complex at the molecular level. The challenges in obtaining crystals of the complex mainly arise from the highly heterogeneous nature of α-DG glycosylation, confirmed by the fact that the protein can be typically visualized in overlay assays or Western blots as a band with a broad smear appearance indicating a distribution of different molecular masses [1, 3, 4].

Recently, Hara and colleagues have extensively analyzed the effect of a missense mutation (T192M) causing the first primary dystroglycanopathy identified so far [18]; nevertheless, the exact molecular mechanism behind this autosomal recessive pathology has yet to be fully clarified. Interestingly, the human T192M mutation (and its topological murine counterpart, T190M, which causes a similar dystrophic phenotype in a mouse model [18]), lies within the strand B1 that together with the strands B2, B3, B4 and B5, arranged in an anti-parallel topology, defines the floor of the basket shaped S6-like domain [13]. Theoretically, the Thr to Met semiconservative mutation is unlikely to cause *per se* a high degree of disorder/instability, and it should not represent a site of O-glycosylation.

Nonetheless, we believe that the structural analysis of the T190M mutant of α-DG is a fundamental step towards the understanding at the molecular level of the mechanism leading to muscular dystrophy. Following this basic idea, we inserted the murine mutant in our recombinant system for expression in *E*.*coli*, and purified it for crystallization studies aimed at possibly solving its structure and comparing it with that of the wild-type protein previously reported [13].

## Materials and Methods

### Mutagenesis and primers

Traditionally, we have worked on murine dystroglycan since it displays a very high degree of identity (93%) with human α-DG. We introduced an additional mutation, R166H, within the N-terminal domain of α-DG in order to make it more resistant to proteolysis [13]; both the wild-type and the mutated variants herein analyzed harbor this mutation. The murine α-DG(50–313)R166H (hereinafter WT) DNA was cloned into a bacterial vector, pHis-Trx, for the expression of the protein as thioredoxin fusion product, also containing an N-terminal 6xHis tag and a thrombin cleavage site, as previously described [13]. The point mutation T190M was introduced into the WT DNA construct using the QuikChange site-directed mutagenesis kit (Stratagene) and appropriate primers. Briefly, the mutation was inserted within the coding region of the WT N-terminal domain cloned in the vector pHis-Trx [13] for prokaryotic expression aimed at crystallographic analysis, and within the entire cDNA sequence of the murine DG cloned in the pEGFP-N1 plasmid for expression in eukaryotic cells [19], respectively, using the following primers:

Forward: 5’-CCAGTGACTGTCCTT**ATG**GTGATTCTGGATGCT-3’

Reverse: 5’-AGCATCCAGAATCAC**CAT**AAGGACAGTCACTGG-3’

The construct DG^T190M^-pEGFP-N1 allows to express DG with a Green Fluorescent Protein (GFP) fused at the C-terminus of β-DG. Moreover, a Myc tag is present, inserted after Lys498, within the C-terminal domain of α-DG [19]. All constructs were verified by automated sequencing.

### Fusion protein expression and purification

The recombinant α-DG(50–313)R166H T190M (hereinafter T190M) fusion protein was expressed in *Escherichia coli* BL21(DE3) Codon Plus RIL strain and purified using nickel affinity chromatography. The fragment of interest was obtained upon thrombin cleavage. Further purification steps were carried out using anion exchange and gel filtration chromatography. Namely, after thrombin cleavage, the flow through of a HiTrap Chelating column (GE Healthcare) was applied onto a Hi-Trap Q HP column (GE Healthcare) pre-equilibrated with buffer A (25 mM Tris–HCl pH 7.5). T190M was eluted with a linear gradient of 0–0.5 M NaCl in buffer A. The fractions containing T190M were pooled, concentrated with Amicon Ultra 15 (Millipore) and loaded on Superdex 200 10/300 GL (GE Healthcare) pre-equilibrated with 25 mM Tris–HCl pH 7.5, 0.15M NaCl, at a flow rate of 0.4 mL/min: the core fractions of the peak were supplemented with 2.5% Glycerol and concentrated by Microcon GM10 (Millipore). The purity of the protein was confirmed by Tricine/SDS-PAGE [20].

The possibility that the methyl sulfide (thioether) of Met 190 would be oxidized in T190M, or in general that the recombinant protein would harbor some additional undesired modifications, has been ruled out via mass spectrometry analysis exploiting an Orbitrap instrument (Thermo Scientific). The corresponding mass value obtained also confirmed the presence of a disulfide bridge between Cys180 and Cys262.

### Crystallization, data collection, structure solution, and refinement

Crystals of T190M were grown by using the vapor diffusion hanging drop method, following the protocol used for the crystallization of WT [13]. Drops were prepared by mixing 1 μL of the protein solution (5.25 mg/mL in 25 mM Tris, 150 mM NaCl and 2.5% glycerol; pH 7.5) with 1 μL of the precipitant solution (0.8 M citrate buffer; pH 7.0) and equilibrated against the reservoir (1 mL) at 4°C.

Data collection was carried out at the European Synchrotron Radiation Facility Grenoble (France), ID23-1 beamline, using a detector Pilatus-6M (Dectris) and the wavelength of 1.00 Å. Data collection was carried out at -173°C. Before being exposed to the X-ray beam, crystals were quickly dipped in cryoprotectant solution (25% ethylene glycol, 1.0 M citrate buffer, pH 7.0) and flash frozen in liquid nitrogen.

Indexing, integration and data reduction of the diffraction data were carried out by using a combination of XDS [21] and CCP4 [22] programs. Data reduction statistics are reported in Table 1.

**Table 1:** X-ray diffraction: data collection and model refinement statistics.

**Data Collection**	
Space group	H 3
Unit-cell parameters (Å)	a = 71.879, c = 144.296
Molecules per asymmetric unit	1
Wavelength (Å)	1.00
Resolution (Å)	48.1–1.59 (1.65–1.59)^a^
Total observations	119373
Unique reflections	35504
R_merge_ (%)^b^	4.3 (44.2)
<I/ σ (I)>	15.28 (1.74)
Completeness (%)	95.0 (71.8)
Redundancy	3.4 (2.3)
**Refinement**	
Number of reflections (work-set/test-set)	33720/ 1784
R_work_ ^c^/ R_free_ ^d^ (%)	14.61 / 16.32
Number of non-H atoms	
Protein	1741
Waters	194
Organic (ethylene glycol)	12
Ions (Cl^-^)	1
Average isotropic B factors (Å^2^)	32.9
Protein (main chain)	28.9
Protein (side chain)	35.8
Solvents	39.1
r.m.s. deviation	
Bond length (Å)	0.010
Angle (deg)	1.245
Ramachandran plot	
favored regions (%)	100
allowed regions (%)	0
disallowed regions (%)	0
PDB code	4WIQ

^a^ Values in parenthesis are given for the highest resolution shell

^b^ R_merge_ = ∑_hkl_∑_j_│I_hkl, j_-<I_hkl_>│/ ∑_hkl_∑_j_ I_hkl_, _j_

^c^ R_work_ = ∑_work-set_│F_obs_-F_cal_│/ ∑_work-set_ F_obs_

^d^ R_free_ = ∑_test-set_ |F_obs_-F_cal_|/ ∑_test-set_ F_obs_

The structure solution of T190M was obtained by Patterson search methods with PHASER [23] using the crystal structure of WT as template (PDB id: 1U2C). Restrained refinement of the crystal structure was carried out by using *phenix*.*refine* [24]. The improvement of the initial model underwent a protocol that included a rigid body fitting stage followed by simulated annealing, coordinates and individual B-factors refinement. The subsequent stages integrated a Translation-Libration-Screw (TLS) model parameterization before the individual B-factors refinement. Automatic refinement cycles were alternated with manual rebuilding sessions performed with COOT [25]. Solvent molecules were identified by the automatic water-picking algorithm implemented in *phenix*.*refine* [24]. The overall quality of these automatically picked solvent molecules were manually checked. Multiple conformations were introduced for selected side chains during the last cycles of refinement.

Protein stereochemistry was monitored throughout the refinement process and during manual rebuilding with MolProbity [26]. Statistics of the crystallographic refinement are reported in Table 1.

Protein structures superposition was carried out using ProFit (Martin, A.C.R., http://www.bioinf.org.uk/software/profit/). The STRIDE web-server was used to assign the protein secondary structure [27]. Interfaces between domains, solvent accessible areas and solvation energies were analyzed by using PISA as part of the CCP4 package [28]. Waters-protein interactions were analyzed using the WAP web-server [29].

Coordinates and structure factors have been deposited in the PDB, with accession code: 4WIQ. Figures were prepared using PyMol [30].

### Fluorescence analysis

Equilibrium fluorescence titrations were performed in 50 mM Tris HCl, pH 7.4, with spectra collected at each guanidine hydrochloride (GdnHCl) concentration (point by point, as to avoid photo-bleaching phenomena) at 25°C in a 1-cm quartz cuvette using a fluorescence spectrophotometer Cary Eclipse (Agilent Technologies). The final concentration of protein (both WT and T190M) was 0.1 μM, and for each point spectra were taken after 20 minutes of equilibration time [31]. The excitation wavelength was 280 nm, and emission spectra were recorded between 300 and 400 nm. The fluorescence peak change as a function of GdnHCl concentration was fitted as described elsewhere [31]; calculations were carried out using the Kaleidagraph software. All the reagents used were of high purity grade from Sigma.

### Limited proteolysis

WT and T190M at 30 μM in 50 mM Tris (pH 8) buffer were subjected to limited proteolysis at 37°C by the addition of trypsin (Sigma) to a final concentration of 2 μg/mL. The reaction was stopped after 1, 5, 10, 20, 40 and 60 min by adding SDS sample buffer to aliquots of the reaction mixture. The samples were analyzed by performing 15% SDS-PAGE and Coomassie staining. As a control the same time course was performed in the absence of the enzyme.

### Super Resolution Microscopy with 3D SIM

Human osteosarcoma U2OS cells cultured in DMEM, 10% FBS, were seeded on No. 1.5H precision cover glass (Marienfeld-Superior). The cells were transfected with the corresponding pEGFP-N1 vectors expressing full-length DG, WT and T190M, with polyethylenimine (PEI) precipitates based on a 3:1 ratio of PEI (μg) to total DNA (μg). 48h post transfection the cells were fixed in 4% PFA.

Lipid staining was achieved after 30 minutes incubation in a 1:2000 dilution of Cellmask Plasma Membrane Orange dye (Invitrogen). For the immunostaining of the Myc-tag fused to DG, cells were permeabilized for 90 seconds in 0.5% Triton X100 and washed in PBS. Cells were blocked in 5% BSA in PBS for 1h followed by an incubation with 1:1000 dilution of primary rabbit polyclonal anti-c-Myc antibody (C3956 from Sigma-Aldrich) in PBS 0.1% Tween-20 for 1h at room temperature. Secondary goat anti-rabbit antibody conjugated to AlexaFluor647 (A-21244, Life Technologies) was applied at a 1:400 dilution for 1h after several washes in PBS 0.1% Tween-20. The cells were finally washed three times in PBS. The cover glass was mounted in Vectashield containing DAPI (Vector Laboratories) for nuclear counter stain and sealed with nail polish.

U2OS cells expressing WT-EGFP and T190M-EGFP were imaged on a 3D Structured Illumination Microscope (3D-SIM) OMX v.4 (GE Healthcare). The refraction index of the immersion oil was chosen to avoid spherical aberrations in the green emission channel. DAPI was excited with a 405 nm, EGFP with 488 nm, Cellmask Plasma Membrane Orange with 568 nm and goat anti-rabbit antibodies bound to AlexaFluor647 with 647nm laser, while sCMOS cameras recorded the corresponding emission in the following bandwidth of 436/31 nm, 528/48 nm, 609/37 nm and 683/40nm. The 3D structured illumination images were reconstructed in the Softworx v6.1.1 software (GE Healthcare) at a Wiener filter setting of 0.004 and using one week old recorded OTFs optimized for the 528 nm emission channel. For presentation here, the final images were adjusted linearly in intensities with the software package Fiji [32] to minimize any visible spherical aberrations in the blue and red emission channels. Raw data are available upon request.

## Results

### Crystallization and crystal structure determination

Well shaped crystals of T190M grew in 7–10 days as regular slabs that diffracted X-rays.

**Fig 1:**
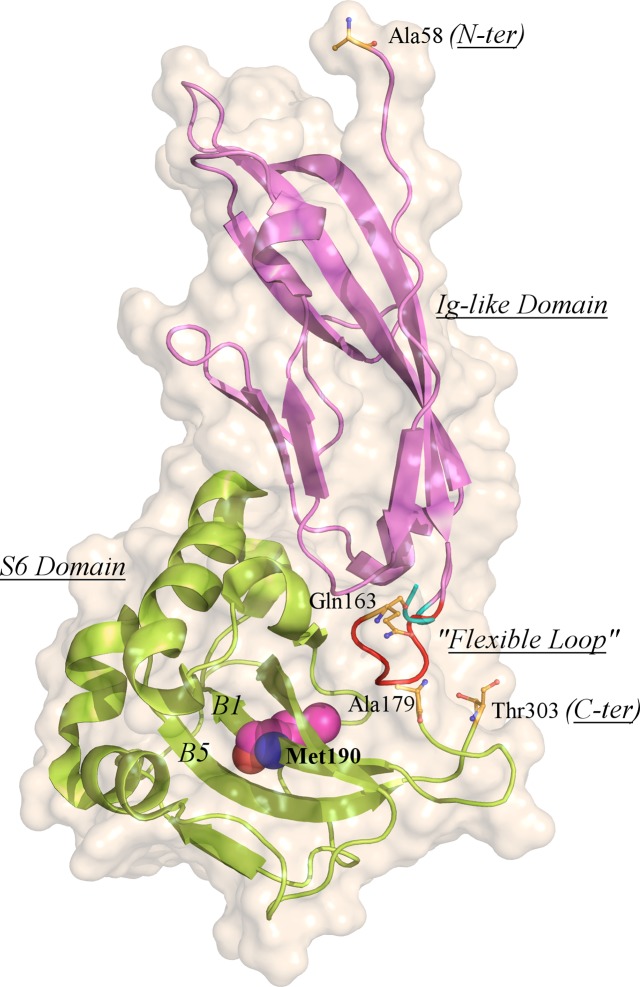
Cartoon depiction of the T190M crystal structure. The two domains are colored in violet (Ig-like domain) and green (small ribosomal S6 subunit domain), respectively; the B1 and B5 β-strands are labeled for clarity. Spheres representation is employed for highlighting Met190. The loop regions connecting the two domains are shown for both WT (colored in cyan) and T190M (colored in red). Residues Ala58 and Thr303 (N- and C-term residues) together with Gln163 and Ala179 (N- and C-term of the modeled loop in the T190M crystallographic structure) are highlighted as stick-and-ball representation.

The crystal structure of T190M has been determined and refined up to a resolution of 1.6 Å. The final model includes residues (58–163) and (179–303), that could be traced in the 2F_o_-F_c_ electron density map. The overall structure of T190M does not show global differences with respect to WT. The root-mean-square deviation (RMSD) between the T190M and WT structures is 0.758 Å (calculated on 268 Cα), which decreases to 0.297 Å when omitting from the RMSD calculation the 4 residues (157–160) being part of the flexible loop discussed in the next paragraph. As reported for WT [13], the structure of T190M consists of two domains (Fig 1): the first one described as an Ig-like domain (residues 60–158) and the second one (residues 180–303) similar to the small subunit ribosomal protein S6. The spatial organization of the domains is conserved in the T190M crystal structure as well as the interaction surfaces between the two domains. A flexible loop, which links together the two domains, could be traced only at the N-terminal side (residues 159–163: His-Asn-Glu-Pro-Gln). It should be noted that the WT structure refers anyway to a protein carrying the additional single mutation R166H that was originally introduced to stabilize the recombinant protein during purification [13]. The same mutation is also present in T190M, although this position falls into the region of the flexible loop that could not be traced in WT as well as in T190M (see below).

**Fig 2:**
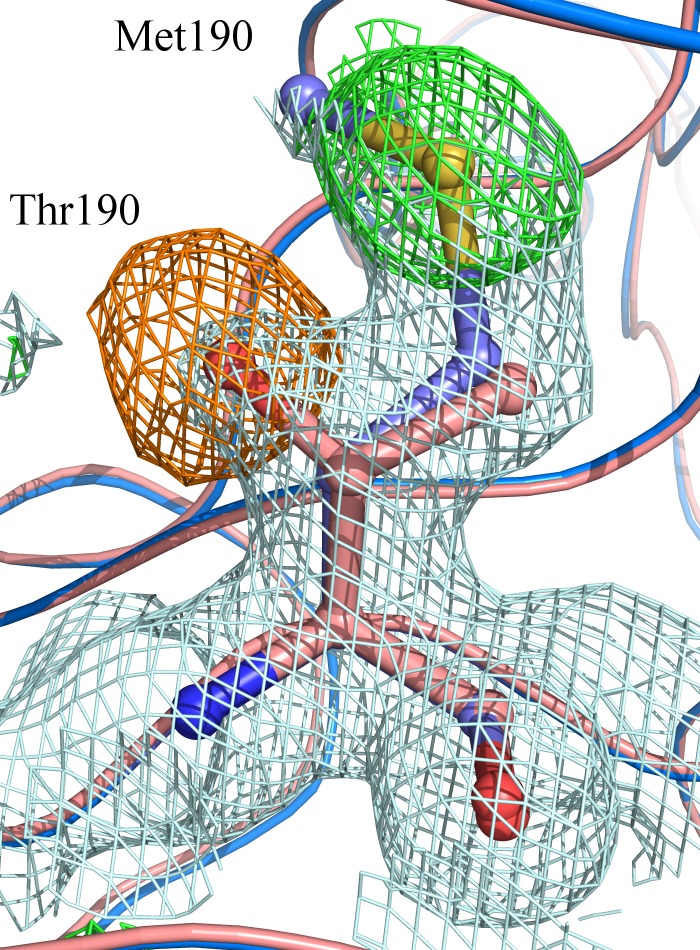
Calculated σ_A_-weighted 2F_o_-F_c_ map (contoured at 1.0 σ and colored in light-blue) and F_o_-F_c_ map (contoured at ± 3.0 σ and colored in green for positive values and in orange for negative values). The maps have been calculated following the Patterson search stage using the WT as a model (PDB id: 1U2C). The final model of T190M (colored in blue) is superposed to the initial Patterson search solution (colored in pink-salmon). Both models are represented as ribbon except residues at position 190 (Thr for the template structure and Met for the mutated final model), which are represented as sticks.

The crystallographic analysis confirmed the substitution of Thr190 with Met, as showed in Fig 2 and, according to the refined model, the T190M mutation does not introduce significant conformational changes in the structure. These findings altogether indicate that this mutation does not significantly alter the overall fold of T190M with respect to that of WT.

**Fig 3:**
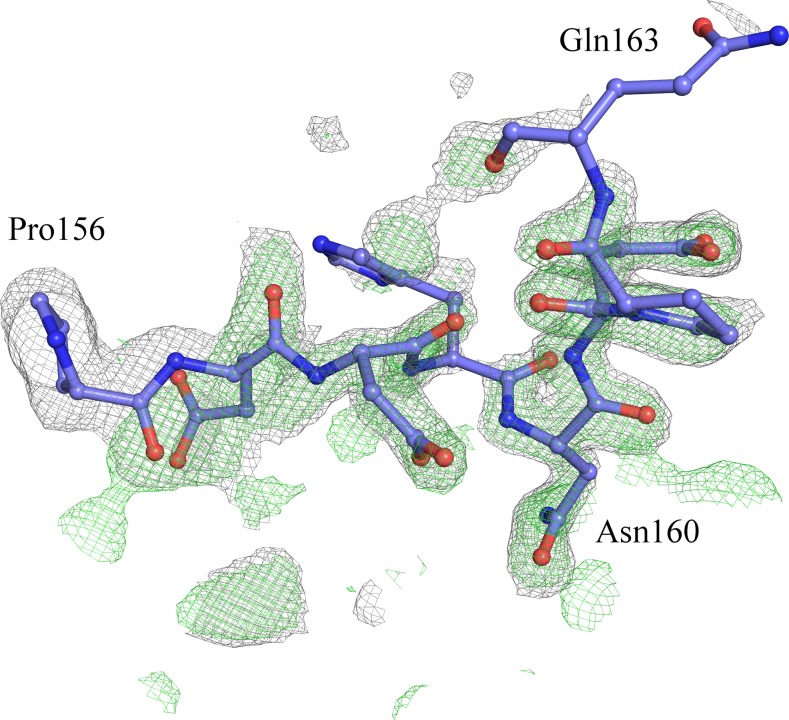
SA-omit σ_A_-weighted 2F_o_-F_c_ map (contoured at 1.0 σ and colored in grey) and F_o_-F_c_ map (contoured at 3.0 σ and colored in green) overlaid with the loop region encompassing residues (156–163) as the T190M final model (PDB id 4WIQ). Maps have been calculated using the T190M model, where residues (156–163) have been omitted from the calculation. The loop of the T190M final model nicely fits the 2Fo-Fc maps, which is indicative of the soundness of the built model.

**Fig 4:**
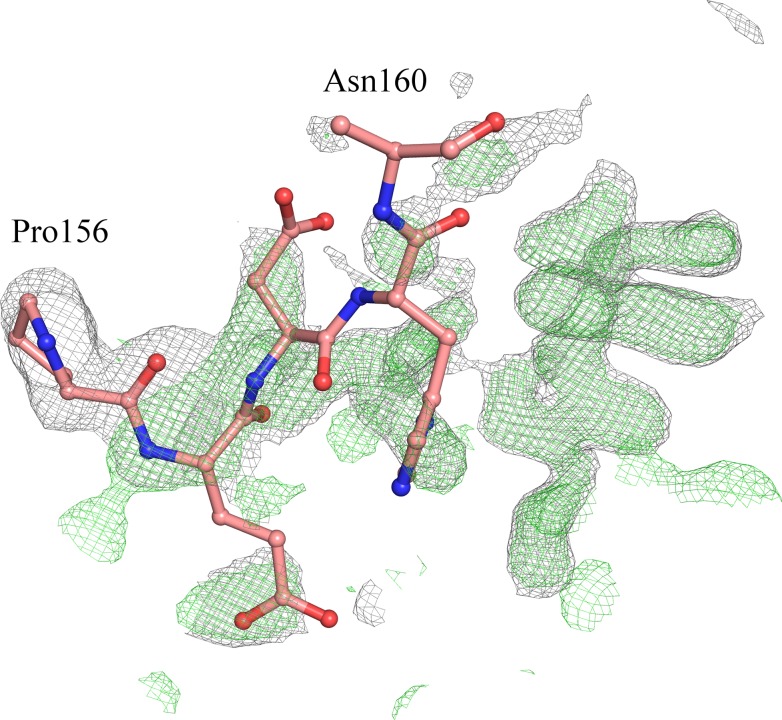
SA-omit σ_A_-weighted 2F_o_-F_c_ map (contoured at 1.0 σ and colored in grey) and F_o_-F_c_ map (contoured at 3.0 σ and colored in green) overlaid with the loop region encompassing residues 156–160 as in the WT model (PDB id 1U2C). Maps have been calculated by using the T190M model where residues (156–163) have been omitted from the calculation.

**Fig 5:**
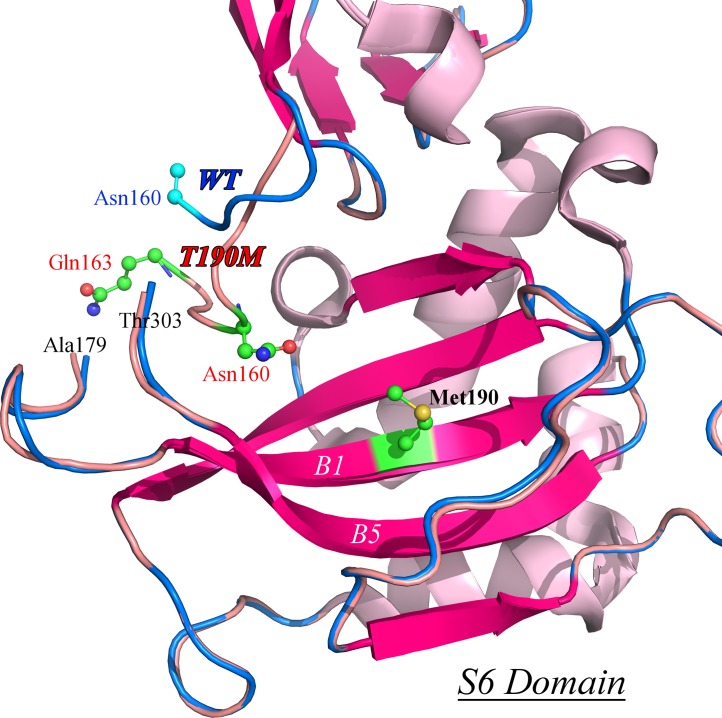
Superimposition of the WT and T190M mutant S6 domains. The models are represented as ribbons, with WT colored in pink-salmon and T190M colored in blue; Met 190 is highlighted in green and depicted as a ball-and-stick model. The “flexible loop” C-term residues Gln163 (T190M) and Asn160 (WT and T190M) are also depicted as ball-and-stick models (Asn160 residue is partially modeled in 1U2C). B1 and B5 β-strands are white labeled for clarity. The superimposition of the two crystal structures emphasize the different conformations of the respective loops encompassing residues (157–163).

Despite showing very similar crystal structures, WT and T190M differ in the conformation of the flexible loop linking the two domains (residues 159–179). Indeed the conformation of the residues (158–163) in T190M, that has been best modeled based on the Fourier maps (Figs 3 and 4) and by monitoring the R_free_, differs with respect to the one being observed for residues (158–160) in WT (PDB id: 1U2C). The refined structure thus suggests that the main-chain loop conformation has changed in T190M with respect to WT. Moreover, in T190M the loop could be traced for three additional residues, up to Gln163, with respect to the WT structure, with residues (158–161) assuming a β-Turn structure (Figs 3 and 5). Being the WT and the T190M crystallization conditions almost identical and the crystal structures exhibiting the same packing features, it is reasonable to correlate the observed differences in the loop conformation to the Thr *vs* Met mutation, which would result in a less flexible loop and in an increased proximity of the loop to the side chain of the mutated residue.

Residue 190 is located at the center of the B1 β-strand, at the bottom of the basket-like domain and is solvent exposed [13]. The point mutation Thr to Met does not introduce drastic local changes in the structure. The Met residue nicely superposes on the position occupied by Thr in the WT (Fig 2), with the Cβ-Cγ bond rotated by ≈ 20° with respect to the corresponding bond (Cβ-Cγ2) in Thr, while the Cγ-Sδ-Cε group is pointing towards the solvent region. Also the nearby residues geometries are not greatly affected by the point mutation.

Despite the limited effect of the Thr to Met mutation on the neighboring residues, the introduction of a bulkier and apolar group such as the methylthio group of the methionine causes a re-organization of the surrounding water molecules. An analysis made with PISA [28] shows that the solvent exposed surface is more than doubled when considering Thr in the WT (≈ 13 Å^2^) and Met in the T190M mutant (≈ 34 Å^2^). Furthermore, the solvation energy contribution estimated by PISA for each residue in the respective crystallographic models goes from -0.01 kcal/mole (Thr in WT) to 0.74 kcal/mole (Met in T190M), indicating a potentially perturbing effect of the methionine on the surrounding water molecules.

**Fig 6:**
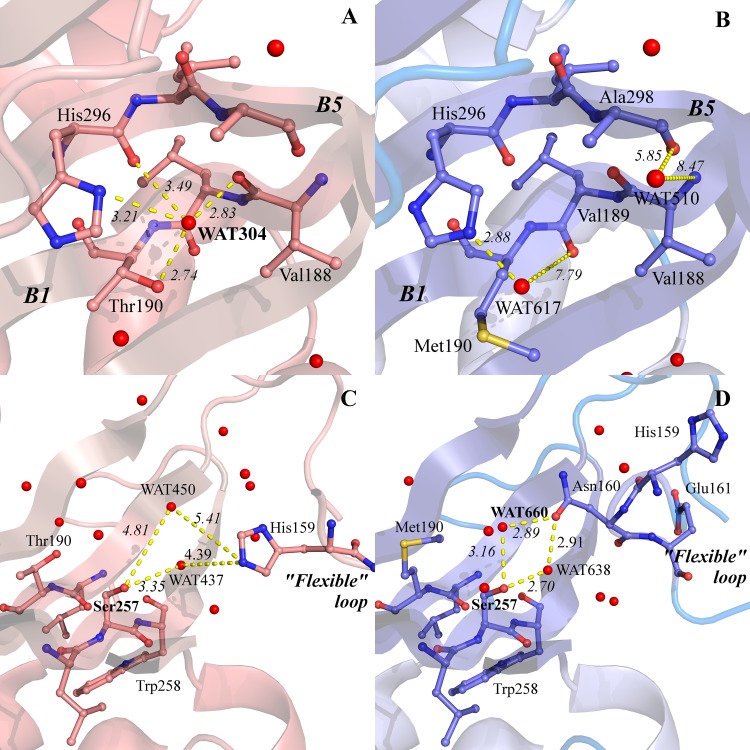
Details of the hydrogen bond networks involving selected water molecules and residues. The structural elements of the N-terminal domain of α-DG are represented as ribbon; residues involved in the interaction networks are represented as sticks. Interatomic distances are reported in Å. The color scheme for WT and T190M models is the same as in Fig 5. A) hydrogen bond network involving WAT304, Thr190 and Val188 in WT, with the B1 and B5 β-strands labeled for clarity. B) The T190M model is shown in the same orientation as WT. Water molecules less than 8 Å apart from a putative WAT304 are also shown. C) WT hydrogen bond network involving Ser257 and the “flexible loop”. Water molecules less than 8 Å apart from a putative WAT660 (water numbering as in PDB id: 1U2C) are also shown. D) T190M hydrogen bond network involving Ser257, WAT660 and WAT638. The T190M model is shown in the same orientation as WT. The comparison of the mutated T190M with WT highlights the differences existing in their solvent structures and emphasizes their role in the interplay between protein structural elements.

An analysis of the solvent structure in the crystallographic models shows that while several waters surrounding residue 190 are conserved in both WT and T190M, some differences in the solvent organization are actually evident (as shown in the four panels of Fig 6). Namely, WAT304 (WT, PDB id: 1U2C) interacts with Thr190 OH in WT but it is absent in T190M (Fig 6A and 6B). The lack of WAT304 in T190M is rather interesting, as this water molecule bridges together the B1 and B5 β-strands by interacting with the backbone of residues Val188 (O) and His296 (O), and with the side chains of residues Thr190 (Oγ1) and His296 (Nδ1). WAT304 acts as hydrogen bonds donor towards residues Val188 (O) and His296 (O) and as hydrogen bonds acceptor from Thr190 (Oγ1) and His296 (Nδ1) (Fig 6A). Such hydrogen bonds are not observed in T190M due to the lack of a bridging solvent molecule that would stabilize the interaction between the B1 and B5 β-strands (Fig 6B). Moreover, solvent structure changes were also found in a region occupied in T190M by Asn160, whereas the same space is populated by solvent waters in WT (Fig 6B). Indeed, Asn160, which is part of the flexible loop discussed above, protrudes from the loop towards Met190 acting, through Oδ1, as an acceptor of bifurcated hydrogen bonds fromWAT660 and WAT638 water molecules (T190M, PDB id: 4QWI); Met190 in turn interacts, still through hydrogen bonds, with Oγ of Ser257, and with other solvent molecules (Fig 6C). In the WT crystal structure WAT660 is not present and WAT638 does not mediate any interaction between the flexible loop and Ser257 (Fig 6D).

It is quite apparent that the substitution of a hydrophilic residue such as threonine with a bulkier and apolar one like methionine results in a solvent re-organization around the residue itself. Changes in the structure solvation sphere may have significant consequences on the conformation of the (159–179) loop. Altogether, the loop relocation and the modified solvation sphere may affect the protein stability and, eventually, also the mutual orientation of the two domains in solution.

### Stability measurements

**Fig 7:**
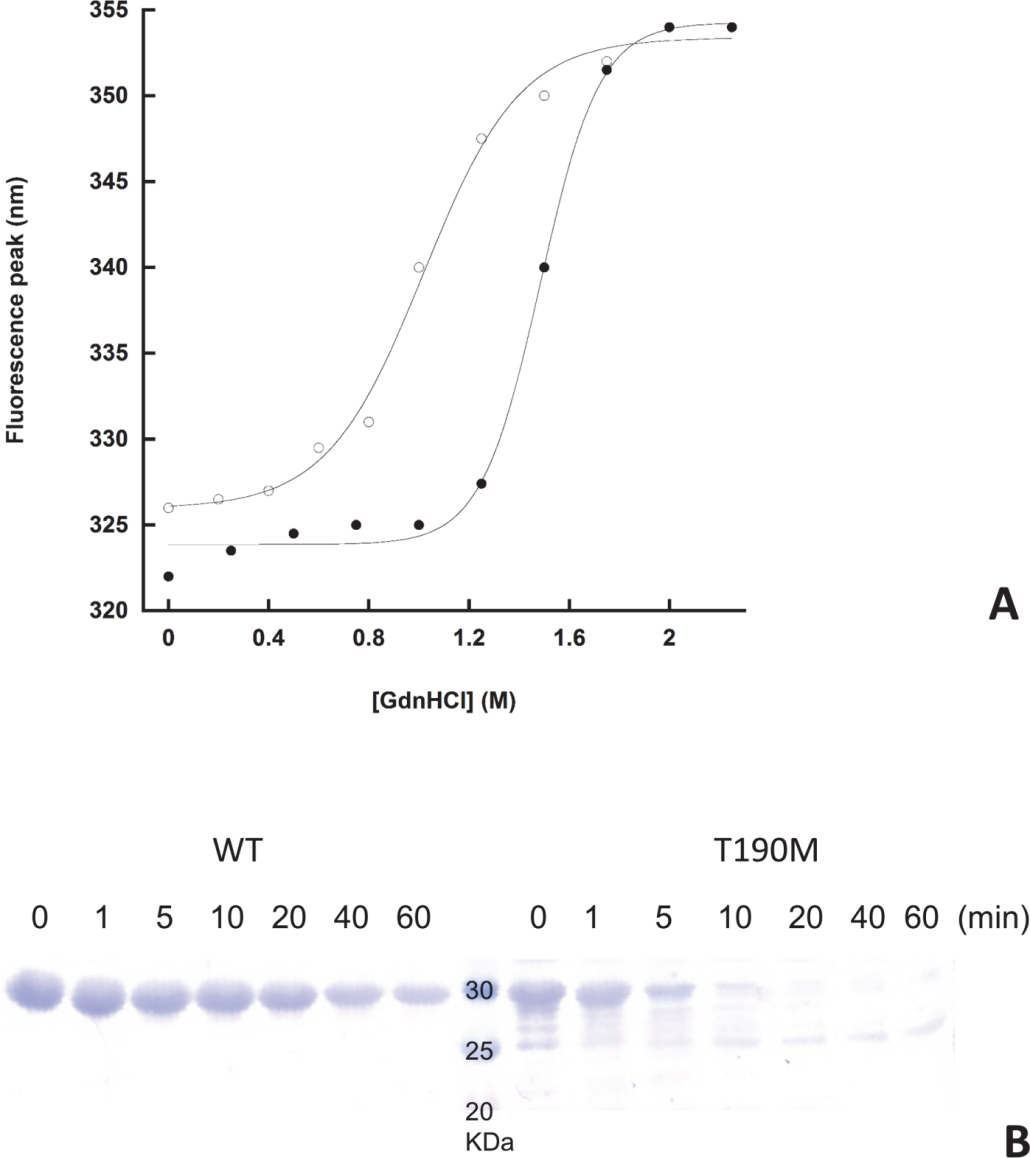
Relative stabilities of WT and T190M. A. Equilibrium unfolding curves of WT and T190M. The red-shifts in the fluorescence peaks upon addition of GdnHCl indicate a progressive exposure of the tryptophan residues to the solvent: the data fit to a single cooperative transition for both WT, closed circles, and T190M, open circles, with a difference in midpoints (Gdn_**50%**_) of 0.45M, that reveals the mutant to be less stable than the wild-type protein. Both experiments were performed in triplicate, and no significant deviation from the above curves was detected. B. Limited proteolysis of WT and T190M. The tryptic digestion time course of the two variants is shown. The faint band(s) below the main band of full length T190M are likely to represent some contaminants and/or the result of a minor degradation event taking place during the purification steps.

Chemical unfolding experiments have been performed in order to establish the relative stabilities of WT and T190M in solution. Fluorescence emission has been used as a mean to record the exposure of the aromatic residues (mainly Trp) to the solvent, with emission maxima that range from ≈ 310 nm of the fully buried to ≈ 350 nm of the fully exposed tryptophan residues [33]. The four tryptophan residues in the WT primary sequence are good fluorescent probes of the unfolding behavior of the domain and its mutant upon addition of a chemical denaturant such as GdnHCl. Fig 7A shows the unfolding curves of WT and T190M as a function of GdnHCl concentration. In the absence of denaturant, the emission spectra of both WT and T190M are those of native-like proteins, with peaks in the spectral region of buried tryptophan residues; the fact that the mutant’s peak is slightly red-shifted as compared to the wild-type might reflect an increased exposure of one or more of its tryptophan residues to the solvent. The plot of the emission spectra peaks as a function of GdnHCl reveals a cooperative unfolding behaviour for both WT and T190M, with transition midpoints (Gdn_50%_, i.e. the denaturant concentration inducing 50% of the total peak shift) of 1.45M and 1M, respectively, indicating that WT is more stable than T190M.

To gain further insights into the stability of the two variants of the α-DG N-terminal domain, limited proteolysis experiments have been performed. Both variants contain 19 predicted potential sites for trypsin cleavage (Peptide cutter tool at http://web.expasy.org) and therefore are likely to be completely degraded following trypsin digestion. T190M was rapidly cleaved by trypsin and completely degraded within 10 minutes of digestion (Fig 7B). WT instead was less susceptible to tryptic cleavage and showed a clear degradation only after 40 minutes of digestion (Fig 7B) but was still present after 1 hour of digestion. Considering that neither of the two variants is subjected to degradation in the absence of the protease in the same experimental conditions, T190M is clearly more susceptible to proteolysis than WT, confirming the latter to be more stable, in accordance with the results of the chemical denaturation experiments.

### Super Resolution Microscopy

The Osteoclast cell line U2OS has been transfected both with the EGFP plasmids carrying the WT and T190M full-length DG variants [19], respectively and the cells have been analyzed by 3D structured illumination microscopy on an OMX v.4 system.

**Fig 8:**
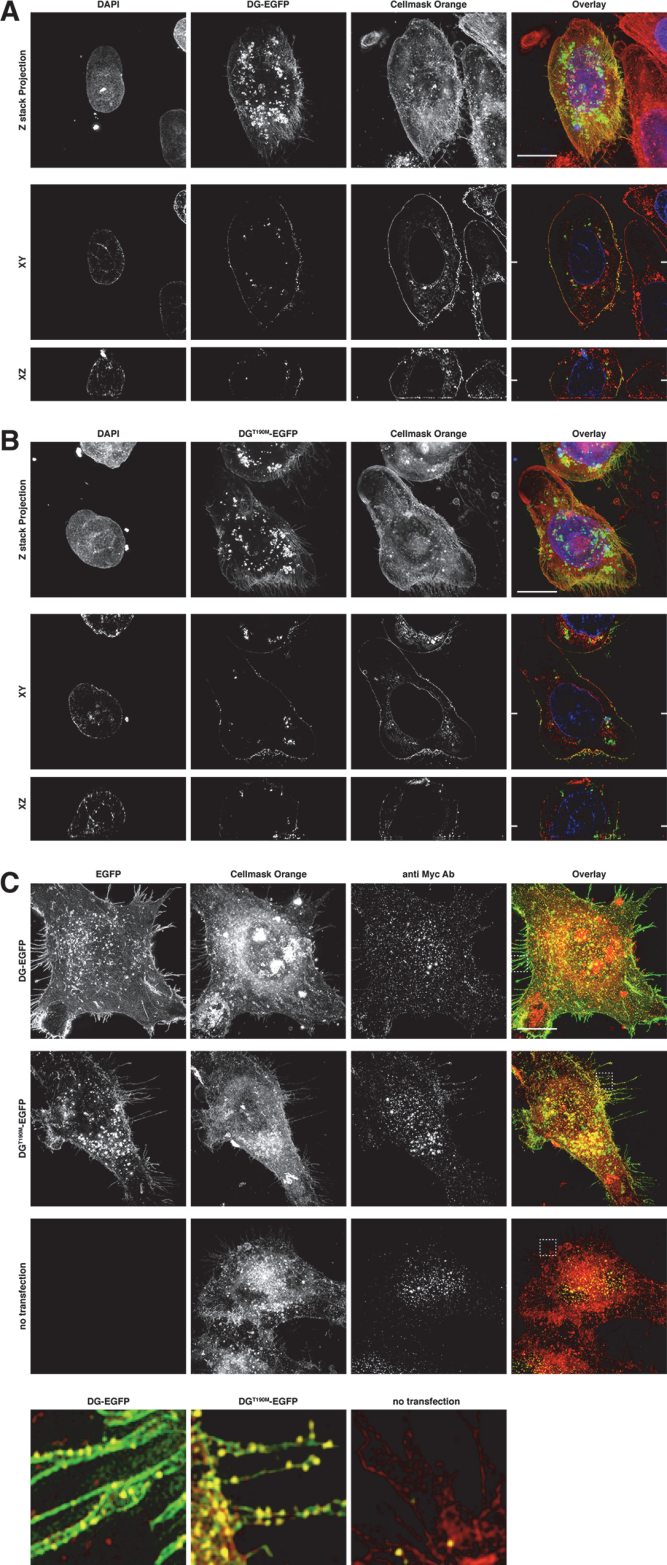
3D Structured Illumination Microscopy images of U2OS cells expressing DG and DG^T190M^ fused to EGFP. DG-EGFP (A) and DG^**T190M**^-EGFP (B) are both localized specifically at the plasma membrane and accumulate in the cytoplasm. The columns show the individual images for DAPI (blue), EGFP (green), Cellmask Plasma Membrane Orange (red) and the resulting RGB overlay. The first row of images is the z stack maximum intensity projection of the 3D recording of the cell. The two subsequent rows of images are at a specific z position in the stack (XY) and X position slice through the stack (XZ). The white bar represents 10μm. Immunolabelling of Myc-tagged Dystroglycan (C) shows a specific signal at the plasma membrane. The individual maximum intensity z-stack projection images for the EGFP, Cellmask Orange and anti Myc channels are shown as well as an overlay. Four by four μm magnifications of the corresponding white dashed boxes depict the Myc-tag signal at the outside of the plasma membrane (filopodia). The antibody stain appears nicely shifted toward the outside of the plasma membrane, while GFP and Cellmask orange are overlapping.

No relevant differences have been observed between WT and T190M, either following the EGFP signal reporting β-DG localization (Fig 8A and 8B) or the anti-Myc reporting α-DG localization (Fig 8C). In both variants, DG undergoes its maturation cycle and it is extensively trafficked *via* the endoplasmic reticulum to the plasma membrane where it is nicely localized to filopodia and also to podosomes located at the cell periphery. Notably, **β**-DG colocalizes with the plasma membrane stain while the anti-Myc α-DG signal appears slightly shifted at the outside of the cell, suggesting no dramatic alterations as far as the distribution of the two DG subunits is concerned.

## Discussion

According to the high resolution X-ray crystal structure here presented, the overall structure of the N-terminal domain of α-DG is not significantly affected by the T190M mutation, nor remarkable local conformational changes of the residues close to position 190 have been detected. At large, this finding seems to be fully supported by the fluorescence microscopy analysis, which does not record dramatic differences in the phenotype of T190M transfected osteoclasts with respect to their WT counterpart. The outcome of the fluorescence microscopy experiments (showing that both DG subunits have a typical localization pattern in T190M) is definitely indicating that the mutation is acting in quite a subtle and intriguing molecular regulatory fashion, or anyway that its effect would be much less observable in an isolated cellular context than in skeletal muscle fibres [18].

The major effect of the T190M mutation seems to be related to the disruption by the bulkier and apolar methionine side chain of the surrounding solvent network structure. Indeed, it is well known that solvation may play a critical role on the stability and the conformational equilibrium of a macromolecule [34], as well as on the protein-ligands interactions [35]. Furthermore, it has been reported that even a single mutation at the surface of a protein, can significantly affect the folding/unfolding free energies as a result of altering the solvent organization [36]. In T190M the differences observed in the solvent organization around Met190 may account for the structural and thermodynamic differences observed with respect to WT. Indeed, T190M resolves in the formation of a new hydrogen bonding network involving Asn160 and the water molecule WAT660, which may influence the flexibility of the 159–179 loop. Moreover, the loss of stabilizing interactions between the B1 and B5 β-strands due to the absence of WT WAT304, may play a role in the T190M lower stability revealed by the chemical denaturation and limited proteolysis experiments (see below).

Indeed, a particularly interesting aspect seems to be that in this case part of the flexible loop encompassing residues 160–180 that in the WT domain was even less structured, due to its high mobility and flexibility [13], assumes in T190M a different conformation. Such a result leads to hypothesize that the Thr to Met mutation could have an impact on the structural dynamics of the entire N-terminal domain. The enhanced rigidity of T190M may affect the overall dynamics and orientation of the two subdomains within the L-shaped α-DG molecular architecture, eventually influencing its global conformation and its function.

This possibility seems to be corroborated by the evidence that in solution T190M has a lower stability than the WT, as suggested by the different melting points calculated on their unfolding curves. Although the T190M denaturation curve has a melting point at about 1 M GdnHCl, denoting a rather stable protein, the decrease in stability with respect to WT is small but significant, as further confirmed by limited proteolysis experiments.

### Potential consequences of T190M/T192M on the DG-LARGE connection

The exact molecular mechanism leading to hypoglycosylation of α-DG carrying the T190M (T192M in human) mutation and causing a severe dystrophic phenotype, accompanied by some degree of intellectual impairment, is currently unknown [18]. *Via* co-immunoprecipitation experiments, it was shown by Hara and colleagues that the mutated N-terminal domain of α-DG is likely to exhibit a reduced affinity towards LARGE. This glycosyltransferase is responsible for adding a repeating disaccharide unit [-α3-GlcA-β3-Xyl-], that is thought to be crucial for extracellular matrix protein binding, to O-mannosylated α-DG [18]. Therefore, it is reasonable to hypothesize that the degree of local perturbation and/or reduced stability measured in T190M in solution could affect the conformation of the N-terminal domain of α-DG, influencing its binding to LARGE and provoking, at least in some cell types or tissues, a significant hypoglycosylation of α-DG.

Kanagawa and colleagues have shown that the N-terminal domain of α-DG is shed by furin, in a process that is likely to take place into the intracellular Golgi compartments in which the final steps of the DG maturation process are completed [37]. An alternative chronological order dictating the chaotic molecular events taking place in the Golgi could imply that such furin-driven enzymatic step would even anticipate LARGE binding to α-DG. In which case, it is reasonable to postulate that such an autonomous domain of α-DG [12] may represent not only a recognition substrate but also an (allosteric?) activator for LARGE [38].

Interestingly, following the findings of a recent computational docking study, it was hypothesized that the T190M/T192M mutation might affect directly the binding of α-DG to laminins [39]. However, we believe that such a mechanism would be highly unlikely. Although we previously measured in the recombinant WT the presence of some residual binding activity towards laminin-1 [13], the estimated affinity was lower than that observed for full-length and glycosylated native α-DG binding to laminin and agrin [5,11,40]. In addition, such residual binding activity was apparently harbored by the first Ig-like domain of α-DG and not by the S6-like domain where the T190M/T192M mutations resides [13]. α-DG post-translational *O*-glycans decorations along the endoplasmic reticulum and modifications accomplished in the Golgi, are crucial for its ability to function as an extracellular matrix (EM) receptor and namely to bind to EM proteins such as laminin, perlecan, agrin and neurexin in the brain and pikachurin in the retina [5]. Notably, the same are required for the binding of α-DG to Old World arenaviruses [16]. Recently, a β-1,4-glucuronyltransferase, designated B4GAT1, has been also identified to be involved in the initiation of the LARGE-dependent enzymatic process [41,42].

Although a few glycosylation sites have been proposed and/or localized within the N-terminal and C-terminal domains of α-DG, most of the O-linked oligosaccharide chains protrude from Thr and Ser residues placed within its central mucin-like domain [2,43,44]. A thorough biophysical characterization of the “naked” mucin-like domain of α-DG clearly pointed to the dramatic role played by the glycosylation in its conformational stability [45].

Unraveling the molecular mechanism of disease induced by T190M/T192M is likely to be rather complex and additional hypothesis can be formulated. For example, the unveiled structural basis for the recognition of an O-glycan, GalNAc-Neu5Ac, and its attached mucin-like peptide GPATPAP by the paired Ig-like type 2 receptor α [46] suggests that the Ig-like domain within the N-terminal α-DG region could function as a protein “hub” for the recognition of the oligosaccharides protruding from the neighboring α-DG mucin-like domain. Furthermore, the same domain could act as a direct docking site for one of the two domains of the glycosyltransferase LARGE [47], belonging to the GT-8 and GT-49 families respectively [48]. Indeed, two mutations hitting the N-terminal Ig-like domain have been recently reported in a patient with hypoglycosylated α-DG [49].

Possibly, in solution, the effect of the T190M mutation, which is next to the flexible hinge connecting the two subdomains, may “propagate” to the Ig-like domain by changing its relative orientation with respect to the S6-like domain also influencing its binding properties. It is worthy of note that the presence of a second Ig-like domain within the C-terminal region of α-DG has been confirmed by bioinformatics analysis and molecular modeling; the domain has been biochemically characterized and shown to include the β-DG binding epitope [50].

### Conclusions

Such interesting structural hypotheses necessarily await further rounds of experimental/computational validations by means of techniques being able to explore the dynamical behavior of α-DG (NMR, Molecular Dynamics). Furthermore, molecular modeling and docking tools will be exploited to investigate the likelihood of α-DG binding to LARGE or to other regulatory factors important for DG maturation. The present structural analysis of the N-terminal T190M variant of α-DG may pave the road to the elucidation of the molecular mechanism of a specific case of dystroglycanopathy, with important consequences for clinicians and for designing possible therapies, but in addition it may be also relevant from a cellular and molecular biology perspective, helping to improve the overall picture of the multiple structural aspects underlying the maturation and function of the DG complex.
